# Identification of LOC101927355 as a Novel Biomarker for Preeclampsia

**DOI:** 10.3390/biomedicines10061253

**Published:** 2022-05-27

**Authors:** Reyna Peñailillo, Lara J. Monteiro, Stephanie Acuña-Gallardo, Felipe García, Victoria Velásquez, Paula Correa, Pilar Díaz, Patricia P. Valdebenito, Cristina Navarro, Roberto Romero, Mario Sánchez, Sebastián E. Illanes, Gino Nardocci

**Affiliations:** 1 Laboratory of Reproductive Biology, Center for Biomedical Research and Innovation (CIIB), Universidad de los Andes, Santiago 7620001, Chile; rpenailillo@uandes.cl (R.P.); lmonteiro@uandes.cl (L.J.M.); sacuna@uandes.cl (S.A.-G.); fgarcia2206@gmail.com (F.G.); vpvelasquez@uc.cl (V.V.); 2 Faculty of Medicine, Universidad de los Andes, Santiago 7620001, Chile; pcorrea@uandes.cl (P.C.); mpdiaz@uandes.cl (P.D.); 3 IMPACT, Center of Interventional Medicine for Precision and Advanced Cellular Therapy, Santiago 7620001, Chile; patricia.valdebenitoc@gmail.com; 4 Department of Obstetrics and Gynecology, Clínica Dávila, Santiago 7620001, Chile; 5 Departamento de Ciencias Biológicas, Facultad de Ciencias de la Vida, Universidad Andres Bello, Santiago 8370186, Chile; c.navarro@unab.cl; 6 Perinatology Research Branch, Division of Obstetrics and Maternal-Fetal Medicine, Division of Intramural Research, *Eunice Kennedy Shriver* National Institute of Child Health and Human Development, National Institutes of Health, US Department of Health and Human Services, Bethesda, MD 20892, and Detroit, MI 48201, USA; prbchiefstaff@med.wayne.edu; 7 Department of Obstetrics and Gynecology, University of Michigan, Ann Arbor, MI 48109, USA; 8 Department of Epidemiology and Biostatistics, Michigan State University, East Lansing, MI 48824, USA; 9 Center for Molecular Obstetrics and Genetics, Wayne State University, Detroit, MI 48201, USA; 10 Detroit Medical Center, Detroit, MI 48201, USA; 11 Molecular Biology and Bioinformatics Lab, Program in Molecular Biology and Bioinformatics, Center for Biomedical Research and Innovation (CIIB), Universidad de los Andes, Santiago 7620001, Chile; mesanchez@uandes.cl

**Keywords:** lncRNAs, placenta, RNA-Seq, cellular localization

## Abstract

Preeclampsia, a disorder with a heterogeneous physiopathology, can be attributed to maternal, fetal, and/or placental factors. Long non-coding RNAs (lncRNAs) refer to a class of non-coding RNAs, the essential regulators of biological processes; their differential expression has been associated with the pathogenesis of multiple diseases. The study aimed to identify lncRNAs, expressed in the placentas and plasma of patients who presented with preeclampsia, as potential putative biomarkers of the disease. In silico analysis was performed to determine lncRNAs differentially expressed in the placentas of patients with preeclampsia, using a previously published RNA-Seq dataset. Seven placentas and maternal plasma samples collected at delivery from preterm preeclamptic patients (≤37 gestational weeks of gestation), and controls were used to validate the expression of lncRNAs by qRT-PCR. Six lncRNAs were validated and differentially expressed (*p* < 0.05) in the preeclampsia and control placentas: UCA1 and HCG4 were found upregulated, and LOC101927355, LINC00551, PART1, and NRAD1 downregulated. Two of these lncRNAs, HCG4 and LOC101927355, were also detected in maternal plasma, the latter showing a significant decrease (*p* = 0.03) in preeclamptic patients compared to the control group. In silico analyses showed the cytoplasmic location of LOC101927355, which suggests a role in post-transcriptional gene regulation. The detection of LOC101927355 in the placenta and plasma opens new possibilities for understanding the pathogenesis of preeclampsia and for its potential use as a biomarker.

## 1. Introduction

Preeclampsia is termed as an obstetric disorder characterized by hypertension (≥140/90 mm Hg), together with proteinuria (≥300 mg in 24 h), following 20 weeks of pregnancy [1]. The incidence of preeclampsia is about 2–8% of all pregnancies and it remains one of the leading causes of maternal and fetal morbidity and mortality worldwide [2,3]. Defects in placental development during the early stages of pregnancy, e.g., impairment of uterine spiral artery remodeling and insufficient trophoblast infiltration, are the main risk factors of preeclampsia [3]. Nevertheless, the molecular mechanisms underlying aberrant placental development are not fully understood.

Long non-coding RNAs (lncRNAs) are RNAs longer than 200 nucleotides that regulate gene expression at the transcriptional and post-transcriptional levels, participating in complex molecular mechanisms that involve genetic imprinting, chromatin remodeling, splicing regulation, mRNA decay, and translational regulation [4]. Different studies report that lncRNAs are associated with diverse diseases, including cancer and cardiovascular diseases [5,6]. These functions can be carried out by direct interaction between the lncRNAs and DNA and RNA (through base pairing) as well as with proteins. LncRNAs can be located either in the nucleus or in the cytoplasm, or in both. Unlike mRNAs, lncRNAs must localize to their site of action in order to perform their function. For example, nuclear retained lncRNAs are usually implicated in epigenetic gene regulation, acting as antisense transcripts, enhancer RNAs or scaffolds for transcription factors, or chromatin modifiers [7,8,9,10]. On the other hand, more than one-half of all lncRNAs localize in the cytoplasm and are involved in post-transcription gene regulation by acting, mostly, as decoys for miRNAs [7,8].

Several studies have shown the essential regulatory roles of lncRNA in preeclampsia [11]. Many differentially expressed lncRNAs in the placentas of preeclamptic patients have been reported, which suggests the potential role of lncRNAs in the pathogenesis of preeclampsia and their function in trophoblast cells [12]. RNA-Seq has been a good tool for new transcript discovery associated with preeclampsia. For example, Xiaoju He et al. identified 738 lncRNAs differentially expressed between preeclampsia and control placentas, suggesting that lncRNAs might play a partial, or a key, role in the development of preeclampsia [13]. Jing Tong et al. performed an RNA-Seq in decidual tissue from women with normal pregnancy, early-onset preeclampsia, and late-onset preeclampsia. These results showed 32 lncRNAs differentially expressed in early-onset severe preeclampsia versus normal pregnancy, 53 differentially expressed lncRNAs in late-onset severe preeclampsia versus controls, and 32 differentially expressed lncRNAs in early-onset versus late-onset severe preeclampsia, suggesting that the expression of lncRNAs is associated with the diagnosis of the disease [14]. Currently, there are no clinically available predictive biomarkers for preeclampsia. The analysis throughout transcriptome exploration and the discovery of numerous lncRNAs could help to determine potential biomarkers for preeclampsia to allow the identification of women at risk of the disease.

The current study aimed to identify lncRNAs expressed in the placentas and plasma of preeclamptic women as possible biomarkers of preeclampsia.

## 2. Materials and Methods

### 2.1. Dataset and Differential Expression Analysis

Analyses were performed by using a publicly available RNA-Seq dataset (GSM3147325) from Gene Expression Omnibus (GEO, https://www.ncbi.nlm.nih.gov/geo/query/acc.cgi, accessed on 5 May 2021). We utilized only the control and preeclamptic patients’ datasets for our identification, consisting of 21 and 20 patients, respectively [15].

Reads were aligned to the human reference genome (GRCh38) by using Spliced Transcripts Alignment to a Reference (STAR) software [16]. After alignment, the gene abundance was determined with the HTseq software (https://htseq.readthedocs.io/en/master/) [17] to calculate the raw reads number for each gene. Differentially expressed genes (DEGs) were estimated by using DESeq2 [18] within the SARTools R package [19]. The DEGs with log_2_ fold change ≥ 1 (upregulated) or ≤ −1 (downregulated), both with *p*-values < 0.05, were considered in comparative analysis.

### 2.2. Sample Collection

Samples from pregnancies complicated by preeclampsia and from normotensive controls were collected at the Gynecology and Obstetrics Department of Clínica Dávila in Santiago, Chile. All women enrolled in this study gave written informed consent for the collection of samples and information. This research was approved by the Ethical Scientific Committees of Clínica Dávila and Universidad de los Andes, Chile. Cases included women with a singleton pregnancy who subsequently developed preeclampsia, and the controls included women with a singleton pregnancy without chronic medical conditions or obstetric complications. Preeclampsia was defined as new-onset hypertension (blood pressure  ≥  140/90 mmHg) and proteinuria (≥300 mg in 24 h) at or after 20 weeks of gestation [2]. Placentas (preeclampsia, *n* = 7; controls, *n* = 7) and maternal peripheric blood (preeclampsia, *n* = 7; controls, *n* = 5) were collected at the time of delivery. Briefly, placental biopsies (~1 cm^3^ spanning from the maternal to the fetal surface) were obtained from the placental cotyledon midway between the cord insertion and placental border, avoiding tissues from areas showing placental calcification or infarction and excluding maternal components. Tissue samples were washed in ice-cold PBS 1× buffer, further cut into smaller pieces (~0.1 cm^3^), placed into sterile DNase-free and RNase-free 1.5 mL microfuge tubes containing 1 mL RNAlater (Life Technologies, Carlsbad, CA, USA), and then immediately placed at 4 °C. After a period of 24 h, RNAlater was removed, and samples were stored at −80 °C until further analysis. Blood samples were collected in BD Vacutainer tubes spray-coated K2EDTA (BD Biosciences, San Jose, CA, USA) and kept at room temperature for 2 h, followed by centrifugation at 1500× *g* for 15 min. Plasma fractions were separated and aliquots were stored at −80 °C until further analysis.

Clinical characteristics of preeclamptic and normal pregnancies are summarized in Table 1. Fourteen placentas and plasma samples were collected from the control (seven) and preeclamptic patients (seven) (Supplementary Table S1). Although two fetuses were under the 10th centile at delivery, none of them were considered an IUGR due to the normal placental function evaluated by Doppler during pregnancy.

### 2.3. RNA Extraction

Total RNA was extracted from 14 samples of frozen placental tissue, using TRIzol Reagent (Invitrogen) according to the manufacturer’s protocol. For samples of 350 µL of plasma, TRIzol LS Reagent (Invitrogen, Carlsbad, CA, USA) was used according to the manufacturer’s protocol. RNA concentration and integrity were evaluated with the Nano Drop ND-1000 spectrophotometer and through agarose gel, respectively.

### 2.4. CDNA Synthesis and qPCR

One microgram of RNA was used for reverse transcription with SuperScript (Invitrogen) according to the manufacturer’s instructions. In the case of plasma RNA, the High-Capacity RNA-to-cDNA Kit (Applied Biosystems, Foster City, CA, USA) was used according to the manufacturer’s instructions.

Determination of lncRNA expression in the placenta and plasma was carried out by using Brilliant III SYBR Green qPCR Master Mix (Stratagene, Santa Clara, CA, USA), according to the manufacturer´s instructions, and amplified with the qPCR System Mx3000P (Stratagene, San Diego, CA, USA). RNAs 18S and U6 were used as housekeeping genes for normalization of the placenta and plasma samples, respectively. The expression was quantified by using the 2(^−ΔΔ^Ct) method. Primers’ details are provided in Table 2.

### 2.5. Prediction of Subcellular Localization and Identification of MiRNAs’ Binding Sites for LOC101927355

To identify the potential subcellular localization of the lncRNA LOC101927355, we predicted their locations by using three predictor web tools: DeepLncLoc, Locate-R, and lncLocator [20,21,22]. We compared five subcellular localizations, including cytoplasm, nucleus, cytosol, ribosomes, and exosomes.

For the identification of the miRNA-binding sites of LOC101927355, we used a prediction tool in miRbase (https://www.mirbase.org).

### 2.6. Statistical Analysis

Statistical analysis and graphs were performed with the GraphPad Prism version 7. Statistical significance was defined at *p* < 0.05 for all analyses. The normality was tested by the Shapiro–Wilk test, and, for non-parametric distribution, the Mann–Whitney test was performed.

## 3. Results

### 3.1. Differentially Expressed Genes’ Identification in PE Samples

**Figure 1:**
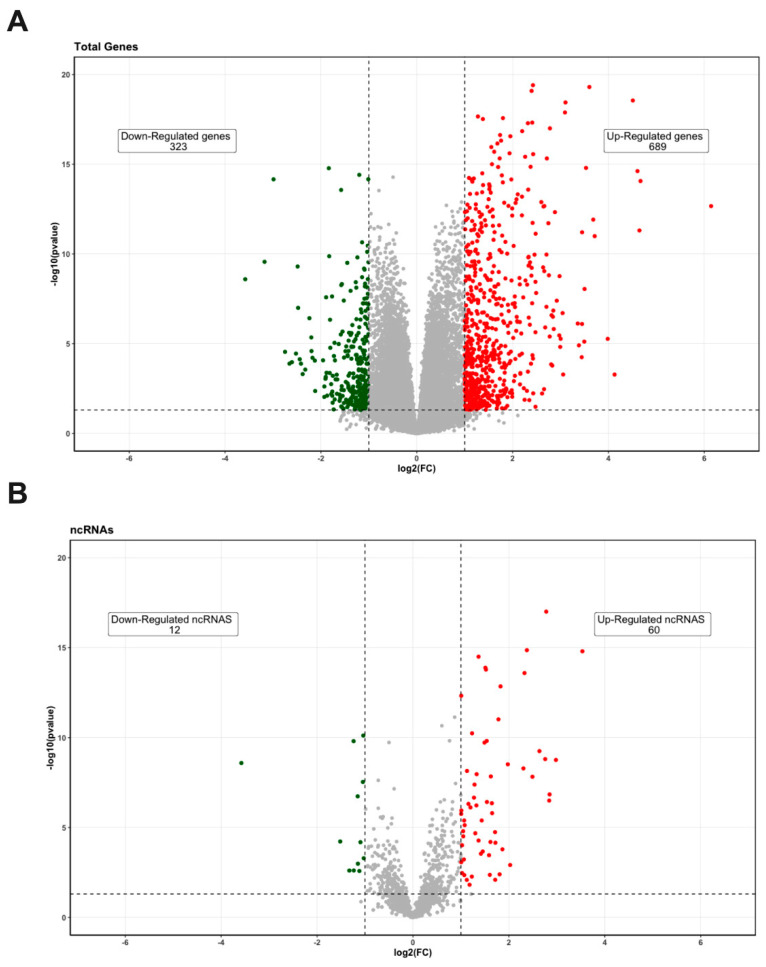
Differentially expressed genes in preeclampsia samples compared with normal pregnancy controls. (**A**) Volcano plot of differentially expressed genes. (**B**) Volcano plot of differentially expressed ncRNAs. The vertical, dotted lines represent log2 fold change ≥1 or ≤−1, and horizontal lines represent *p*-value < 0.05. Red and green spots represent upregulated and downregulated genes, respectively.

**Figure 2:**
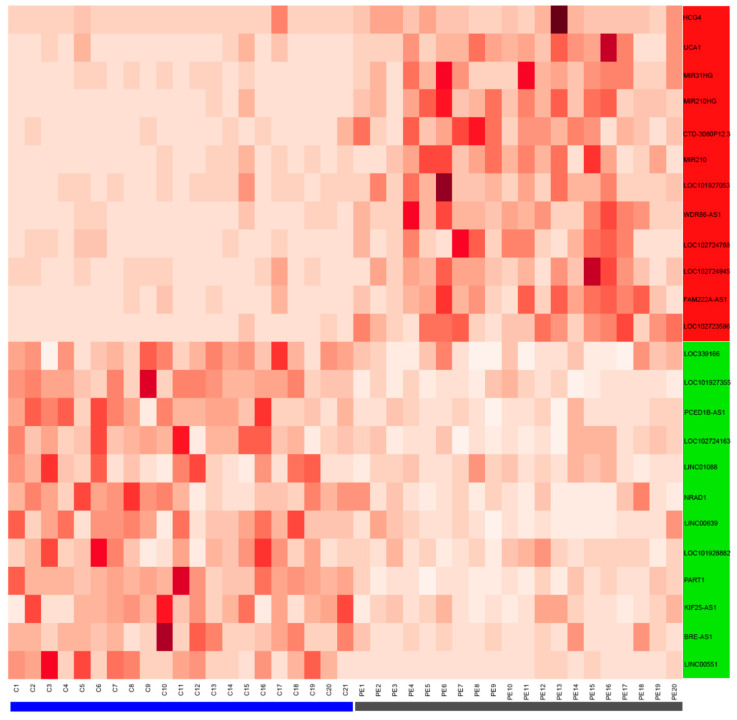
Differentially expressed genes in preeclampsia samples compared with normal pregnancy controls. Heatmap of the top 12 up- or downregulated ncRNAs. The red rectangle indicates upregulated ncRNAs and the green rectangle represent downregulated ncRNAs. Horizontal rectangles denote control (blue) and PE (gray) samples. The normalized counts are represented in the heatmap.

A total of 33,121 genes were analyzed from the dataset GSM3147325 between normal and preeclampsia patients. The data obtained showed a large number of genes that were either upregulated (689) or downregulated (323; Figure 1A). Among them, 60 ncRNAs were upregulated and 12 ncRNAs were downregulated (Figure 1B). The top 12 upregulated and downregulated ncRNAs were analyzed (Figure 2). Only six ncRNAs (UCA1, HCG4, LOC101927355, LINC00551, PART1, and NRAD1) were further analyzed.

### 3.2. Validation of LncRNAs in the Term Placentas of Preeclamptic and Control Patients

**Figure 3:**
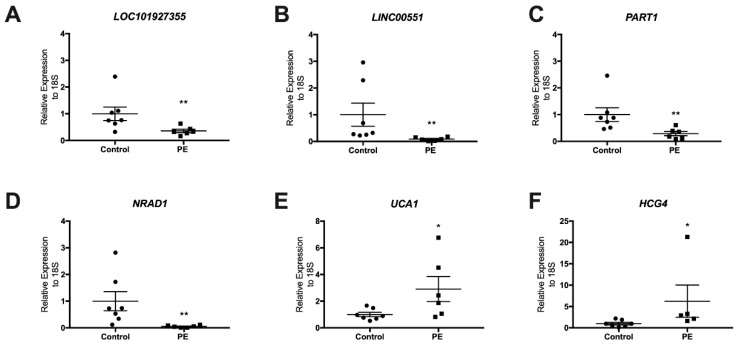
LncRNAs’ expression in term placentas. LncRNAs (**A**) *LOC101927355*, (**B**) *LINC00551*, (**C**) *PART1*, (**D**) *NRAD1*, (**E**) *UCA1*, and (**F**) *HCG4* were validated in seven PE (squares) and seven control (circles) placentas; * *p* < 0.05, ** *p* < 0.01. Mann–Whitney test. The bars indicate the standard deviation.

We identified the expression of six lncRNAs in placental tissues by using qPCR among the seven controls and seven preeclampsia placentas analyzed. As observed in Table 1, there were no significant differences of age and BMI between the preeclamptic and normotensive pregnant women; however, the preeclampsia group showed increased systolic and diastolic blood pressure as well as lower gestational age and birth weight (Table 1). The qRT-PCR results indicated that *LOC101927355*, *LINC00551*, *PART1,* and *NRAD1* were downregulated in the preeclampsia placenta samples (*p* = 0.009, *p* = 0.012, *p* = 0.004, *p* = 0.001, respectively) compared to the control placentas. In the case of the lncRNAs *UCA1* and *HCG4*, their expression was upregulated in the preeclampsia placentas (*p* = 0.035 and *p* = 0.012, respectively) compared to the control group (Figure 3).

### 3.3. Determination of LncRNAs in Maternal Plasma at Delivery

**Figure 4:**
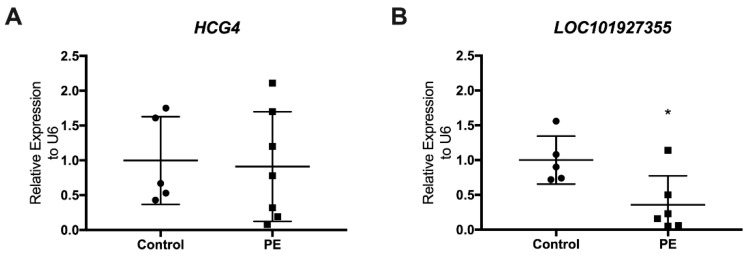
LncRNAs’ expression in maternal plasma. Two lncRNA were detected in maternal plasma at delivery in seven PE (squares) and five control (circles) plasma (**A**) *HCG4* and (**B**) *LOC101927355*; * *p* < 0.05, Mann–Whitney test. The bars indicate the standard deviation.

To explore whether the six placenta-expressed lncRNAs were present at detectable levels in maternal plasma, we utilized RT-qPCR to determine lncRNA expression levels in plasma samples from the same women. Only two of the six lncRNAs expressed in the placenta were identified in maternal plasma at birth: *HCG4* and *LOC101927355* (Figure 4), with the latter showing a significantly reduced expression in the preeclamptic patients (*p* = 0.038) compared to the control group (Figure 4B).

### 3.4. Subcellular Localization of LncRNA LOC101927355

To gain insight into the potential mechanisms of action of lncRNA *LOC101927355*, we performed an in silico analysis of the localization of this ncRNA, since, as mentioned before, subcellular localization of lncRNAs carries essential information for the understanding of their biological functions [23,24]. Using three bioinformatic tools (DeepLncLoc, Locate-R, and lncLocator), we obtained a putative cytoplasmic localization for LOC101927355 [25] (Table 3). This result provided the first clue toward the characterization of the mechanism by which LOC101927355 exerts its function in preeclampsia.

**Figure 5:**
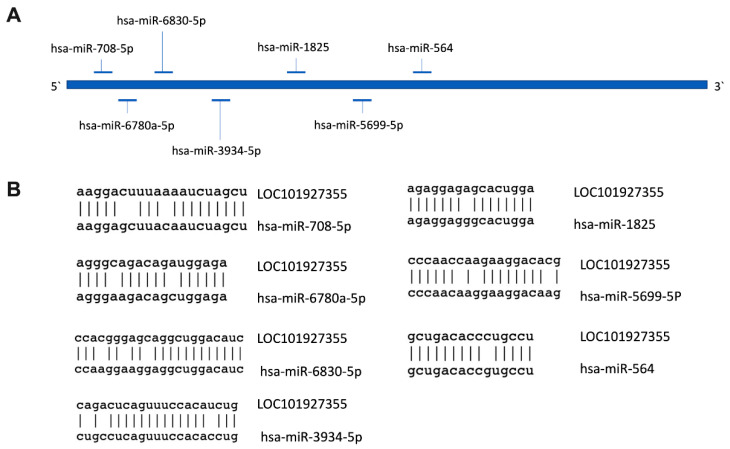
Putative binding sites for miRNAs to the lncRNA LOC101927355. (**A**) Schematic representation of LOC101927355 and the predicted miRs’ target sites; (**B**) alignment of *LOC101927355* to mature miRNAs’ targets.

Cytoplasmic localization can be indicative of lncRNAs to act as an miRNA sponge or to interact with mRNA partners [23]. To evaluate the possibility that LOC101927355 acted as an miRNA sponge, we used an miRNA-binding site prediction tool (miRbase) to determine the miRNA-binding site present in the LOC101927355 transcript. Our analysis found seven putative sites for miRNAs located along LOC101927355 (Figure 5). From these miRNAs, only hsa-miR-708-5p has been related to play a role in placental development [26]. Specifically, this microRNA has a differential expression profile between the first and third trimesters in human placentas.

## 4. Discussion

Aberrant expression of lncRNAs has been detected in the placentas of patients with preeclampsia compared to healthy controls [12,13,27,28] but with scarce information about their presence in the plasma of these patients. Although these noncoding RNAs are not translated to proteins, they play an important role in the modulation of RNA translation. The present study uncovered the downregulation of LOC101927355, LINC00551, PART1, and NRAD1 and the upregulation of HCG4 in the preeclampsia placentas. Further, lncRNA LOC101927355 was downregulated in the plasma of the same women with preeclampsia, suggesting the potential role as a biomarker of preeclampsia. Different studies have suggested that cell-free lncRNAs are detectable in human plasma and may be utilized as minimally invasive biomarkers for disease prediction, diagnosis, and prognosis [29]. However, the main limitation is the quantification of circulating lncRNAs due to the low abundance in the circulation. Early identification of women at high risk of developing preeclampsia would enable surveillance and early intervention, with the potential for drastically improving pregnancy outcomes for the mother and the baby. HCG4 and LOC101927355 lncRNAs could be worthy of further research when seeking novel biomarkers for predicting and monitoring the onset of preeclampsia at the early stages of pregnancy.

Several studies have investigated the role of lncRNAs in the pathogenesis of preeclampsia throughout their function in trophoblast migration, proliferation, and invasion [11]. The lncRNA UCA1 has been widely studied in cancer samples [30,31] and in trophoblast cells [32,33,34]. Our study confirmed what was published previously, where UCA1 expression is increased in preeclampsia placentas [32]. Moreover, studies in trophoblast cell lines highlight its role in cell proliferation, migration, and invasion [32,33,34], essential processes for the correct development of the early placenta and the development of preeclampsia. The remaining lncRNAs analyzed in this study have not been described as related to preeclampsia pathology but share similar functions in cancer cell lines. In fact, the lncRNA LINC00551, which is downregulated in lung adenocarcinoma [35] and esophageal squamous cell carcinoma (ESCC) when silenced, promotes ESC cell proliferation, migration, and invasion in ESCC [36]. Prostate androgen-regulated transcript 1 (PART1) lncRNA can regulate the proliferation and invasion of prostate cancer cells [37] and breast cancer cells [38,39,40]. In the case of LINC00284, also known as non-coding RNA in the aldehyde dehydrogenase 1 A pathway (NRAD1), it is upregulated in ovarian [41,42] and breast cancer cells [43] and also involved in angiogenesis in ovarian cancer cells [41]. The human leukocyte antigen complex group 4 (HCG4) lncRNA is associated with the recurrence of laryngeal cancer [44]. Meanwhile, LOC101927355 lncRNA has not been described previously.

LncRNAs can affect gene expression in multiple ways, and their localization could provide some insights about their role in the cell. Our analyses predicted that LOC101927355 is located in the cytoplasm with the possibility to act as an miRNA sponge due to the miRNA-binding sites found within its sequence by the prediction tool. Cytoplasmic lncRNAs can influence gene regulation by acting as decoys for miRNAs and proteins. Among the predicted miRs’ target sites, we found hsa-miR-708-5p that acts as an oncogene to promote cell proliferation, migration, and invasion [26,45], which are crucial processes during placentation. Moreover, this miRNA is highly expressed in first-trimester human placentas in comparison to third-trimester placentas, and therefore associated with placental development [26].

Among the limitations of our study, we reported the reduced number of available samples, including the missing plasma samples of two control patients. Moreover, prospective cohort studies are required to determine the early expression of LOC101927355 and HCG4 lncRNAs in the maternal plasma. It is also a limitation that we did not match both groups for gestational age. Indeed, the possibility of having age-matched controls is complex since deliveries under 37 weeks of gestation usually have pregnancy complications.

In the future, a bigger cohort study is required to determine the expression of LOC101927355 lncRNA in the maternal plasma in early pregnancy to confirm its value as a biomarker. As preeclampsia it is a multifactorial disease, we understand that a single biomarker cannot deliver the required prognostic performance to identify early pregnant pre-symptomatic women. Combining multiple biomarkers into a predictive test is an approach that achieves higher diagnostic and prognostic test performance and should be further evaluated.

## 5. Conclusions

The detection of differentially expressed lncRNAs in the placenta and plasma of patients with preeclampsia opens new possibilities not only for understanding the biological mechanisms underlying the pathogenesis of preeclampsia but also suggests their potential use as biomarkers. The lncRNA LOC101927355, decreased in the placenta and maternal plasma at delivery, could therefore represent a novel potential biomarker for preeclampsia. Nevertheless, to assess its potential as a new biomarker for preeclampsia, we need to validate these findings in a larger cohort with plasma samples collected during the first half of the pregnancy, i.e., before the development of preeclamptic symptoms.

## Figures and Tables

**Table 1:** Clinical characteristics of the study population.

Characteristics	Control (*n* = 7)	Preeclampsia (*n* = 7)	*p*-Value
	Mean ± SD	Mean ± SD	
Age (years)	34.8 ± 6.3	29.6 ± 4.0	0.1358
BMI (kg/m^2^)	31.5 ± 2.2	30.6 ± 3.2	0.9825
Systolic blood pressure	113.8 ± 10.2	153.4 ± 27.9	0.0006
Diastolic blood pressure	66.2 ± 7.6	88.3 ± 14.3	0.0017
Gestational age at delivery	38.4 ± 1.0	33.14 ± 4.4	0.0006
Birth weight	3551.0 ± 442.7	1953.6 ± 768.4	0.0006

BMI: Body mass index. Birth weights are not corrected by sex.

**Table 2:** List of primers for quantitative polymerase chain reaction used in this study.

Gene	Sequence
*18S Fw*	GCCGCTAGAGGTGAAATTCTTGGA
*18S Rev*	ATCGCCGGTCGGCATCGTTTAT
*U6 Fw*	CTCGCTTCGGCAGCACA
*U6 Rev*	AACGCTTCACGAATTTGCGT
*LOC101927355 Fw*	CTCTGACTCTGTATTTCAGGAAGC
*LOC101927355 Rev*	TTGTGGTAAAGGGAGATAGGAAGG
*LINC00551 Fw*	GGATTTGGAAGAACAAACGGG
*LINC00551 Rev*	GGTCAAATACTCTGGTAGCTCC
*PART1 Fw*	GTGATCTGGGGAAAACGCA
*PART1 Rev*	GGGAATCGGTTGTGAGTAGG
*NRAD1 Fw*	ATGTGAGTGATCAGTAACACC
*NRAD1 Rev*	GAACCACGAAGACAAGGAT
*UCA1 Fw*	GGCCCTCATTCCGTGAAGAG
*UCA1 Rev*	CTCCACCGTAAGAGTTACCCGA
*HCG4 Fw*	CCAGGGAGAAACCCTCGGAAT
*HCG4 Rev*	AAACCCTGTCTCTACACCTCCATT

**Table 3:** Subcellular localization tools.

Predictor	Nucleus	Cytoplasm	Cytosol	Exome	Ribosome	Predicted Location
DeepLncLoc	0.330	0.447	0.090	0.017	0.116	Cytoplasm
Locate-R	0.06	0.85	-	0	0.09	Cytoplasm
lncLocator	0.154	0.799	0.022	0.0087	0.0140	Cytoplasm

## Data Availability

Not applicable.

## Supplementary Materials

The following supporting information can be downloaded at: https://www.mdpi.com/article/10.3390/biomedicines10061253/s1, Table S1: Clinical Characteristics of the Study Population for each patient.
