# Supplementary material for: Identification of LOC101927355 as a Novel Biomarker for Preeclampsia
Source: Biomedicines. 2022 May 27;10(6):1253. doi: 10.3390/biomedicines10061253 (PMC9219905; doi:10.3390/biomedicines10061253)
Supplement: Supplementary file 1 [file biomedicines-10-01253-s001.zip › biomedicines-1720547-supplementary.pdf]

Supplementary Table S1. Clinical Characteristics of the Study Population for each patient.

Patients	Systolic Blood Pressure	Diastolic Blood Pressure	Gestational Age at Delivery	Birth Weight (g)	Percentile	Proteinuria (mg)	BMI
C1	95	55	38	3665	90	0	27.5
C2	124	75	38	3570	75	0	34.2
C3	112	64	38	4055	90	0	33.6
C4	126	61	38	3380	75	0	31.6
C5	112	76	40	3085	10	0	30.5
C6	112	67	39	4420	90	0	30.9
C7	116	70	40	3500	50	0	31.6
**Average**	**113.8**	**66.2**	**38.4**	**3551.0**		**0**	**31.5**
PE1	140	80	36	2915	50	dipstick 2+	25.3
PE2	138	82	37	2230	<10	429	32.0
PE3	170	110	37	2740	25	1338	34.5
PE4	130	90	34	1540	<10	dipstick 2+	27.3
PE5	210	105	27	1065	75	1140	30.8
PE6	140	71	27	1000	50	594	32.4
PE7	146	80	34	2185	25	dipstick 2+	32.0
**Average**	**153.4**	**88.3**	**33.14**	**1953.6**		**506.14**	**30.64**

BMI: Body mass index. Birth weights are not corrected by sex.
